# The Role of the Tumor Microenvironment in T-Cell Redirecting Therapies of Large B-Cell Lymphoma: Lessons Learned from CAR-T to Bispecific Antibodies

**DOI:** 10.3390/cancers17020317

**Published:** 2025-01-20

**Authors:** Kirill V. Lepik, Vladislav V. Markelov

**Affiliations:** RM Gorbacheva Research Institute of Pediatric Oncology, Hematology and Transplantation, Pavlov University, 191144 St. Petersburg, Russia

**Keywords:** tumor microenvironment, TME, large B-cell lymphoma, LBCL, chimeric antigen receptor T-cells, CAR-T, bispecific antibodies, BSAs, T-cell redirecting therapies, immune checkpoints

## Abstract

T-cell redirecting therapies, which include chimeric antigen receptor T-cells (CAR-Ts) and bispecific antibodies (BSAs), have revolutionized the treatment of relapsed\refractory large B-cell lymphoma (LBCL). Expanding clinical experience with these advanced therapies shows the potential for the optimization of their use with combination or consolidation strategies, which necessitates the prognostic stratification of patients. While traditional clinical prognostic factors identified in the era of chemotherapy are characterized by limited value, the tumor microenvironment (TME) is becoming a new prognostic cluster. We examine how the heterogeneity of LBCL, characterized by variations in tumor parameters and differences in TME immune cell composition, immune checkpoint expression, and cytokine milieu, correlates with both positive responses and resistance to treatment. While classical parameters such as histological subtype, cell of origin, and target antigen expression lack proven prognostic value for T-cell redirecting therapies, the density and functional state of tumor-infiltrating lymphocytes, tumor-associated macrophages, and immune checkpoint molecules are shown to be critical determinants of therapeutic success, particularly in CAR-T therapy. We identify several gaps in the current knowledge and suggest that the insights gained from CAR-T experience could be instrumental in refining BSA applications. This report also highlights limitations in the current knowledge, as TME data derive from a limited number of registrational trials with varying methodologies, complicating cross-study comparisons and often focusing on immediate response metrics rather than long-term outcomes. By dissecting the complex interactions within the TME, this review aims to identify new prognostic factors and targets, ultimately fostering more effective and tailored treatment strategies for LBCL patients.

## Simple Summary

Large B-cell lymphoma (LBCL) is a type of blood cancer that can be challenging to treat, especially when it does not respond to standard chemotherapy or relapses after treatment. New therapies, such as CAR-T cells and bispecific antibodies, have shown promise in treating these cases. However, not all patients benefit equally from these treatments, highlighting the need for new ways to predict treatment response. This study focuses on the tumor microenvironment, which includes the immune cells and molecules surrounding the LBCL tumor cells. By understanding how this environment affects CAR-T and bispecific antibody treatment outcomes, this manuscript aims to identify factors that can help predict success or resistance to therapy. These insights could lead to more personalized treatment approaches and improved outcomes for patients with LBCL.

## 1. Introduction

Large B-cell lymphoma (LBCL) represents a heterogeneous group of aggressive non-Hodgkin lymphoma (NHL) subtypes, predominantly afflicting adults and characterized by the rapid growth of transformed large B-cells. Diffuse large B-cell lymphoma, not otherwise specified (DLBCL NOS) being the most common subtype, accounts for approximately 30% of all the NHL cases, with a median age at diagnosis of around 70 years [1]. The cornerstone of DLBCL treatment has traditionally been rituximab in combination with cyclophosphamide, doxorubicin, vincristine, and prednisone (R-CHOP), reflecting the standard first-line therapy [2]. With the current standard, about 25–35% of the patients exhibit primary resistance or relapse post-treatment, with even higher rates of treatment failure for patients with higher-risk LBCL types, such as high-grade B-cell lymphoma (HGBL) or DLBCL NOS with aggressive disease features [3]. A significant number of randomized trials exploring alternative therapeutic approaches have failed to surpass the efficacy of R-CHOP, with a notable exception of the Pola-R-CHP regimen incorporating polatuzumab vedotin, which, however, did not solve the problem of relapsed and refractory (r/r) LBCL [4]. Outcomes for r/r LBCL patients remain dismal, with limited therapeutic options and poor overall survival rates in the era of chemotherapy [5]. In the second-line setting, autologous stem cell transplantation (ASCT) following salvage chemotherapy has historically represented a critical therapeutic option. The efficacy of this approach is limited, with approximately 25% of the patients eligible for intensive chemotherapy achieving prolonged remissions [6]. Unfortunately, for patients who are not candidates for transplantation or those who fail salvage chemotherapy, the prognosis was even more discouraging [5].

T-cell redirecting therapies are a form of immunotherapy implemented by targeting T lymphocytes to a pre-selected antigen expressed on the surface of tumor cells through the genetic modification of T-cells or pharmacological effects, which holds significant promise in this context. The main types of T-cell redirecting therapies currently used in clinical practice are T-cells with chimeric antigen receptors (CAR-Ts) and bispecific antibodies (BSAs) [7].

CAR-T is an innovative approach in which T-cells are genetically modified ex vivo with a transgene encoding a chimeric receptor [8]. In the products approved to date, chimeric receptors combine in their structure the antigen-binding fragment of immunoglobulin and the signaling part of the T-cell receptor with the domain of immune cell costimulatory receptors. This approach allows CAR-T to recognize the target independently of a major histocompatibility complex (MHC), and also to induce target-specific cytotoxicity without additional activating signals [8]. Currently, five commercial anti-CD19 CAR-T are approved for the treatment of r/r LBCL in the third and subsequent lines of therapy: tisagenlecleucel (tisa-cel), axicabtagene ciloleucel (axi-cel), and lisocabtagene maraleucel (liso-cel) in USA and Europe; actalycabtagene autoleucel (actaly-cel) in India; and relmacabtagene autoleucel (relma-cel) in China [9,10,11]. In this group of patients, the overall response rates (ORRs) with anti-CD19 CAR-T ranged from 53% to 83%, and complete remission (CR) rates ranged from 39% to 83%. The median overall survival (OS) and progression-free survival (PFS) reported in registration clinical trials were 11.1–25.8 months and 2.9–6.8 months, respectively [10,12,13,14,15]. Encouraging results of CAR-T in a group of highly pre-treated patients have led to research into the effectiveness of CAR-T at earlier lines of treatment. Tisa-cel, axi-cel, and liso-cel were compared in randomized trials with high-dose chemotherapy followed by ASCT, representing the standard of care for the second-line treatment of LBCL. Axi-cel and liso-cel demonstrated a significant benefit in OS and event-free survival (EFS) compared with the control group, which served as the basis for the US and European regulatory approval for the use of these CAR-Ts in the second-line therapy of LBCL [16,17,18].

The mechanism of action of approved bispecific antibodies (BSAs) involves one antigen-recognition domain binding to a target on the surface of the tumor cell, while the other domain binds to CD3, a molecule expressed on T-cells as part of the T-cell receptor complex. This interaction leads to the formation of an immune synapse between the malignant cells and the T lymphocytes, inducing T-cell-mediated cytotoxicity independent of T-cell receptor/MHC. Currently, numerous anti-CD3/anti-CD20 BSA are being tested in clinical trials for the treatment of LBCL in third-line or subsequent therapy lines with glofitamab, epcoritamab, odronextamab, and mosunetusumab being the most developed to date [19,20,21,22]. The first three on this list have demonstrated excellent activity in patients with r/r LBCL in corresponding pivotal trials with the observed ORR ranging from 52% to 63% and CR rates from 31% to 39%. At a two-year follow-up, the median OS with odronextamab therapy was 9.2 months, while in the studies with glofitamab and epcoritamab, the median OS was not reached. The median PFS was 4.4 months [19,20,23]. Based on the results of phase II studies, glofitamab and epcoritamab were approved for patients with LBCL who have received two or more lines of therapy, while odronextamab is undergoing regulatory review [24]. In contrast, mosunetuzumab demonstrated limited efficacy in aggressive B-cell lymphomas [22].

Importantly, the broader clinical experience with CAR-T therapy offers valuable insights that can enhance our understanding of response mechanisms and resistance to BSAs, as these treatment types have biological similarities. First, both approved CAR T-cells and BSAs are derived from monoclonal antibody technology, sharing similar antigen-recognition domains. Besides CD19, CAR-T therapies targeting antigens like BCMA and CD22 have demonstrated significant clinical efficacy [25]. These antigens are validated targets for BSA development [8]. Second, as the mechanisms of T-cell signaling and the implementation of effector functions have much in common in both types of redirecting therapies, as discussed in detail in the review by Y. Gao et al. [26], they determine the overlapping profile of complications after CAR-T and BSA, and allow for the use of common approaches to their mitigation [27]. CAR-T therapy experience has led to standardized management protocols for cytokine release syndrome and neurotoxicity, including the use of tocilizumab and corticosteroids, which are directly applicable to BSAs [28].

Both types of T-cell redirecting therapies have the potential to achieve prolonged remissions. An analysis of the long-term results of the ZUMA-1 trial demonstrated that in the total group of patients, 30.3% were characterized by durable remissions [13]. Among the patients treated with glofitamab, 78% with a complete response maintained this status after one year of follow-up. This represents 31% of the overall patient population treated with glofitamab [29]. With both CAR-Ts and BSAs, CR in approximately half of patients is not sustained. Most relapses occur in the first 12 months after the start of T-cell redirecting therapies [12,13,14,15,16,17,18,19,20,21,22]. To prevent relapse in this population of patients, it is necessary to consider consolidation therapy, such as allogeneic HSCT [30]. Importantly, to date, there are no markers or factors that enable the identification of the group of patients with CR who are at high risk of relapse. At the same time, despite the superiority of CAR-Ts and BSAs over chemotherapy for r/r LBCL, between 17% and 47% still remain refractory and in need of additional treatment [12,13,14,15,16,17,18,19,20,21,22].

Therefore, T-cell redirecting therapies should be built into a comprehensive treatment strategy for LBCL, which requires stratification factors. Traditional clinical prognostic factors, such as LDH level, bulky disease, ECOG status, and extranodal involvement are characterized by limited prognostic value [31,32]. The tumor microenvironment (TME) may become a new prognostic cluster, helping to better stratify patients and optimize treatment strategies. However, the impact of TME composition on the prognosis of patients receiving T-cell redirecting therapies, especially BSAs, remains uncertain.

This article provides an overview of the relationships between the structure of the TME and its dynamics, the phenotype of tumor cells, and the profile of immune checkpoints affecting the effectiveness of approved T-cell redirecting therapies in patients with r/r LBCL. It focuses on analyzing the TME parameters evaluated in clinical trials of CAR-Ts and BSAs to identify potential prognostic markers and practical insights that may guide future BSA studies based on CAR-T therapy outcomes.

## 2. The Tumor Microenvironment in Large B-Cell Lymphoma

The tumor microenvironment plays a critical role in the pathology and treatment responses of LBCL. Because of LBCL type heterogeneity, the specific cellular composition varies widely, hampering studies of the microenvironment. LBCL tumor cells alter T-cell and NK-cell function by downregulating MHC molecules or losing the expression of CD58, limiting antigen presentation and cytotoxic effector mechanisms. Additionally, malignant B-cells can express immunosuppressive ligands (e.g., PD-L1) or secrete molecules (e.g., TGF-β) that recruit regulatory T-cells and tumor-associated macrophages and alter stroma, creating an immune-tolerant microenvironment [33]. These tumor-intrinsic strategies disrupt the normal tissue architecture, leaving only a limited presence of TME cells including macrophages, T-cells, NK cells, and stromal cells [33]. Within the TME, immune cells may carry inhibitory receptors like programmed cell death protein 1 (PD1), lymphocyte-activation gene 3 (LAG3), and T-cell immunoglobulin and mucin-domain containing 3 (TIM3) [34]. While these receptors can suppress antitumor immune activity supporting the survival of cancer cells, they also represent important markers for the processes of T-cell activation and exhaustion. The prognostic relevance of certain TME characteristics, like the level of immune infiltration and functional state of T-cells, and the density and polarization of tumor-associated macrophages (TAMs) has been demonstrated for the first-line treatment across different types of LBCL [34,35,36,37,38]. Several recent studies showed that “hot” tumors, specifically those with increased T-cell proportions and expressing PD1 and TIM3, were associated with an improved prognosis for DLBCL patients after rituximab-based chemotherapy, while “cold” DLBCL cases with a depleted TME were associated with significantly worse outcomes [39]. The relevance of the described TME characteristics for the efficacy of different types of T-cell redirecting therapies is an area of active research.

This review focuses on reports of the TME components and their respective prognostic significance for T-cell redirecting therapies. Such components include the following:

Tumor cell characteristics;

Immune cell populations;

Immune checkpoint profile;

Cytokines and chemokines;

Stromal components and adhesion molecules.

## 3. Cell Populations Within the TME

### 3.1. Tumor Cells

Although LBCLs are often grouped as a single entity, they exhibit significant heterogeneity in phenotypic characteristics, pathogenesis mechanisms, and mutational landscapes, which may influence their responses to immunotherapy.

*Histological subtype*: Pivotal clinical studies of CAR-T therapy did not identify the LBCL subtype as a significant factor influencing outcomes. However, real-world data suggest that primary mediastinal B-cell lymphoma (PMBCL) and transformed follicular lymphoma (tFL) may exhibit more favorable responses to CAR-T therapy [31,40,41,42,43,44,45]. It was also demonstrated that patients with a higher “FL-like” gene expression score had higher complete response rates and longer progression-free survival in liso-cel therapy [46]. Similarly, when using BSAs, the LBCL subtype was also not identified as a determinant of prognosis in prospective clinical trials [19,20,21,47]. However, in light of CAR-T data, a more detailed study of this factor in real-life clinical practice with expanded patient cohorts is needed.

*Cell of origin (COO)*: For standard chemoimmunotherapy, the cell of origin (COO) of DLBCL holds known prognostic significance. Lymphomas derived from germinal B-cells (GCB subtype) have a better prognosis than those derived from activated B-cells (ABC subtype) when treated with R-CHOP chemotherapy [48]. However, T-cell redirecting therapies appear to negate the impact of COO on patient outcomes. [19,20,32,40,41,43,46,47,49,50].

*Double-hit\triple-hit lymphomas*: Contrary to standard chemotherapy, where BCL2 and MYC rearrangements predict a poor prognosis, T-cell redirecting therapies have been effective in managing these traditionally unfavorable phenotypes. As shown in a series of reports, neither double-expressor variant of LBCL nor double-/triple-hit lymphoma were prognostic factors among patients receiving CAR-Ts or BSAs [19,31,41,46,49].

*Target antigen expression*: The role of target antigen expression levels on malignant B-cells remains unresolved. The density of the target on the surface of B-cells varies. The expression level of CD20 in patients with DLBCL is higher and less variable than CD19 according to immunohistochemical and molecular genetic studies [51,52]. The JULIET study showed that for tisagenlecleucel treatment, baseline CD19 expression levels assessed by immunohistochemistry did not correlate with response outcomes. Moreover, the ORR was comparable between patients with clearly CD19-positive LBCL and CD19-negative or low-expression lymphoma [41]. However, in two cases where a lack of CD19 expression was observed, responses were not achieved. In the ZUMA-1 study, similar response rates on axi-cel were observed in patients with CD19-negative disease and those who had CD19 positivity according to immunohistochemistry [43,53]. In addition to immunohistochemistry, a transcriptomic analysis of CD19 expression by RNA-seq in patients did not show an impact on either PFS or OS across the overall study population. However, it is worth noting that 6 of 8 CD19 negative patients who initially responded to axi-cel subsequently had disease progression [53]. In contrast, ZUMA-7 EFS after axi-cel was better in patients with high (>median) CD19 gene and protein expression, assessed by immunohistochemistry or gene signatures analysis, compared with patients with lower expression [54]. In the liso-cel study, the levels of CD19 gene expression before CAR-T were comparable between patients with ongoing CR at 3 months and those with disease progression [46].

In studies of the effectiveness of BSAs glofitamab, mozunetuzumab, and odronextamab, no significant association was found between the level of CD20 expression by immunohistochemistry on tumor cells and ORR. However, researchers still pay attention to the fact that patients with extremely low or negative CD20 expression tend to fail to achieve a response to BSAs [47,49,51,55].

In vitro experiments have shown that CAR T-cells are able to eliminate malignant cells that express <100 CD19 molecules on their surface. This number is below the detection limit of immunohistochemistry or flow cytometry [51,56]. Overall, the available data do not show that the expression levels of CD19 and CD20 on B-cells, once above a certain threshold, significantly influence the ORR or other clinical outcomes. However, in situations where the expression of the target is absent or extremely low, although such cases are rare in the LBCL population, T-cell redirecting therapies have little effect and predominantly result in a lack of response or early progression. The discrepancy in the results of the trials may be explained by cases of false CD19 negativity when the expression level of this marker is below the detection limit of the methods used in clinical practice. In anecdotal cases of sustained CR to CAR-T therapy in patients with confirmed CD19 negative lymphoma, the achievement of an antitumor effect may be mediated by mechanisms other than CD19 antigen-dependent cytotoxicity, such as “bystander effect” [41,46,53].

Antigen loss can be considered the ultimate adaptation of a cancer cell to targeted immunotherapy. At relapse after anti-CD19 or anti-CD20 T-cell redirecting therapies, approximately 30% of patients experience target loss. This highlights the higher evolutionary pressure exerted by CAR-Ts and BSAs compared to naked monoclonal antibodies, where the loss of antigen is casuistically rare. In cases of target-positive relapses, other mechanisms come to the fore, with an important role attributed to the influence of the TME components [57].

### 3.2. Tumor-Infiltrating T-Cells

Tumor-infiltrating lymphocytes (TILs) are a population of T-cells, heterogeneous in phenotype and function, forming part of the tumor microenvironment both in the primary and in metastatic sites [58]. In most studies, elevated levels of CD3+ TILs have an impact on the response and prognosis of patients treated with anti-CD19 CAR-T. This underscores the importance of an environment that permits T-cell infiltration, highlighting the role of both genetically modified and non-modified T lymphocytes in the antitumor response [50,59,60,61]. High values of Immunoscore index, which assesses the abundance of CD3+ and CD8+ TIL subsets at tumor loci, and Immunosign 21, which assesses the expression of the set of genes associated in particular with T-cells (*CD3D*, *CD3E*, *CD3G*, *CD8A*, *GZMA*, *GZMB*, *GZMK*, and *GZMM*) were significantly positively correlated with OS in patients with LBCL treated with axi-cel [60,61]. The independent influence of CD8+ and CD4+ T-cell numbers is unclear. The different methods for determining T-cell subsets among studies are also a barrier to identifying their prognostic value. Generally, the density of CD8+ TILs in pre-treatment biopsies had either a neutral or beneficial effect on ORR to CAR-T. The presence of CD8+ TILs expressing PD1+, PD1+ LAG3 +/− TIM3−, or CD73+ was significantly associated with achieving response or prolonged response duration in the patients treated with axi-cel or liso-cel, but not with tisa-cel [61,62,63,64]. Data on the prognostic value of CD4+ TILs are limited. It was shown that the number of T helpers in pre-treatment biopsies did not affect the OS during axi-cel treatment [61]. Patients with persisting response at month 3 after liso-cel had a higher percentage of CD4+ TILs in tumor samples [64].

In BSA therapy, similarly, the enrichment of the TME by TILs creates the prerequisites for achieving remissions. A trend for the higher number of CD3+ and CD8+, but not CD4+ TILs, in lymphoma biopsies before the initiation of odronextamab was observed in patients with response in the ELM-1 trial, but none of these effects reached significance. Surprisingly, the enrichment of the Treg in the TME was associated with response to treatment with odronextamab. Patients who achieved CR to glofitamab also showed a trend toward a higher percentage of CD8+ T-cell infiltration in the TME [49,51]. A positive association was confirmed between the signature of CD4+ follicular T helper genes and response rates to mosunetuzumab in patients with tFL but not DLBCL [65].

Dynamic TME remodeling during T-cell redirecting therapy significantly affects TILs. A number of reports show that the density of TILs after CAR-T or BSAs increases [46,51,59,66]. It was demonstrated that CD3+ TILs are predominantly enriched by CD8+ T-cells. There is limited data regarding the dynamics of changes in other T-cell subpopulations. Notably, the proportion of CAR-positive T-cells varies from 1 to 22% of TILs in the TME. This, again, emphasizes the versatility of the mechanisms of the antitumor response induced by CAR-T. In the patients treated with axi-cel, durable response was associated with a significant increase in T subsets in post-infusion biopsies. These include cytotoxic T-cells, CD4+ naive T-cells, and T helper 2 cells. In the liso-cel trial, patients who achieved an objective response tended to have a greater increase in CD8+ cells within their tumors compared to those who experienced stable disease or progression [46,51,59,61,64,66,67].

To sum up, TME studies show that TILs may serve as valuable prognostic markers for the effectiveness of T-cell redirecting therapies in LBCL. However, inconsistencies in methodologies across studies and limited understanding of the distinct roles of T-cell subsets remain challenges, underscoring the need for standardized methods in future research in both CAR-Ts and BSAs.

### 3.3. Tumor-Associated Macrophages

It is known that the role of TAMs in the microenvironment is ambivalent and is largely determined by their polarization. The M1 polarization of TAMs is induced by Th1 cytokines (IFN-γ, TNF-a, and IL-1b). M1 TAMs provide a pro-inflammatory environment, secrete immunostimulatory cytokines (IL-1, IL-6, IL-12 IL-18, and IL-23) and antiangiogenic factors, present antigens, and support the antitumor response. On the contrary, M2 TAMs are characterized by an anti-inflammatory functional phenotype, produce immunosuppressive cytokines (IL-10, IL-4, IL-13, and TGF-β) and angiogenesis factors, inhibit the antitumor response, cause T-cell dysfunction, and promote the growth and proliferation of tumor cells [68,69].

In published reports, CD68 and CD163 are commonly used to identify TAMs; however, these markers do not indicate their functional polarization. Few studies have examined both the overall population of TAMs and the specific aspects of their polarization. A number of authors have demonstrated that TAM density is associated with refractoriness to anti-CD19 CAR-T therapy. Patients with higher levels of CD68- and/or CD163-positive cells in tumor biopsies had shorter duration of response to axi-cel and lisa-cel [40,61,63]. When using relma-cel, the depth of the response was also determined by the number of TAMs [59] with the negative role confirmed for M2 TAMs. In patients who relapsed after axi-cel, initial tumor biopsies were enriched with M2 TAMs [67].

It has been demonstrated that the density of TAMs increases after CAR-T therapy [46,59,61,64,67]. The available data on the relationship between the dynamics of changes in TAMs and the clinical effect are contradictory. Factors such as TAM polarization (M1 vs. M2 phenotypes), the functional state of macrophages, and interactions with other components of the tumor microenvironment may contribute to these discrepancies.

In the context of BSAs, information on the prognostic value of TAMs is limited. Brouwer-Visser et al. demonstrated that the density of CD68+ TAMs did not differ between responders and non-responders to odronextamab [51]. However, the total number of TAMs alone may not be a sufficient marker, as data from CAR-T trials have highlighted the importance of macrophage polarization. Therefore, in the context of BSA therapy, further research is needed to assess the effects of TAM polarization and dynamic changes in TAM populations.

### 3.4. Myeloid-Derived Suppressor Cells

MDSCs are a heterogeneous population of cells with potent immunosuppressive activity. The origin of MDSCs are the cells of monocytic or granulocytic lineage. If MDSCs are formed from granulocytes, they are called granulocytic/polymorphonuclear MDSCs (PMN-MDSCs), and if from monocytes, then monocytic MDSCs (M-MDSCs). The main parameter defining MDSCs is their ability to inhibit T-cell and B-cell immune responses through the secretion of immunosuppressive cytokines, a number of enzymes, and the expression of ligands for immune checkpoint receptors [70,71].

The data from the ZUMA-1 trial shows that the density of M-MDSCs and PMN-MDSCs in the TME did not differ significantly between complete responders and patients with other types of response to axi-cel [61,72]. However, it is also noted that patients who had a relapse after CAR-T therapy are characterized by an increase in the number of M-MDSCs in post-treatment biopsies [61].

Although the role of MDSCs in the TME appears to be limited for CAR-T therapies, this does not exclude their potential impact on BSAs. Currently, the influence of MDSCs on outcomes in LBCL patients treated with BSAs remains unexplored. Given their known immunosuppressive functions, further research should assess the association of M-MDSC and PMN-MDSC in the TME before and during the treatment with response and prognosis after BSA therapy.

## 4. Immune Checkpoints in the TME

The immune checkpoint family includes more than two dozen members, with notable examples such as PD-1, CTLA-4, LAG3, TIM3, and TIGIT playing a pivotal role in modulating immune tolerance. In contrast, CD28, ICOS, 4-1BB, and CD40 are considered activators of the immune response. Both inhibitory and activating checkpoints, along with their corresponding ligands, are particularly interesting candidates for identification as markers for the success of T-cell redirecting therapies [73].

Due to the clinical importance of PD-1, LAG3, and TIM3, most studies addressed the prognostic significance of these markers on the response and prognosis of patients treated with CAR-T. Using immunofluorescence panels and gene expression profiles, higher baseline presence of PD-1+ T-cells was significantly associated with early CR versus patients who had progression in the first month after CAR-T infusion in the liso-cel TRANSCEND NHL-001 trial [63]. However, only a trend was shown for this factor in a later report for the same cohort [46]. The comprehensive study of the pre-treatment TME profile of the patients treated with axi-cel included a differentiated approach to analyze the isolated or simultaneous expression of immune checkpoints PD-1, LAG3, and TIM3 on CD8+ T-cells. The density of CD8+ T-cells with activated phenotype (PD-1+ and LAG3+/−, TIM3−) was most significantly associated with objective response, contrasting with non-activated (no checkpoint expression) or exhausted phenotype (PD-1+LAG-3+TIM-3+) [61]. In a limited cohort of 10 patients treated with relma-cel, the CR group demonstrated significantly higher relative mRNA expression of *LAG3* and *CTLA4* in pre-treatment tumor biopsies [59]. For tisa-cel, a quantitative immunofluorescence analysis was performed on pre-infusion tumor tissues demonstrating no significant differences between the best overall response groups in the percentage of total cells expressing PD-1, LAG3, or TIM3 and percentage of total CD3 T-cells expressing these markers at baseline. However, patients with the highest percentages of LAG3+ T-cells (among total T-cells) did not have a response to tisagenlecleucel or had a relapse within 6 months [41].

The inhibitory immune checkpoint ligands expression profile in patients treated with CAR-T is less described. The percentages of malignant B-cells (defined as CD19+ and/or CD20+) in pre-infusion biopsy that were also positive for PD-L1 or MHC II were higher in patients with no durable response to axi-cel compared with durable response defined as remission with a minimum follow-up of 6 months after infusion [40]. A subset of CD163+ macrophages, those that were IDO1+ or PD-L1+, appeared to be higher in the pre-infusion samples of patients with no response to lisa-cel [46]. For tisa-cel, no significant influence was shown for the percentage of total cells expressing PD-L1 in pre-infusion biopsies to best response. However, the 5 patients with the highest PD-1–PD-L1 interaction scores either did not have a response to tisa-cel or had an early relapse [41].

Several studies addressed the influence of activating immune checkpoint expression. Some of the strongest individual genes with higher expression in the pre-infusion samples of patients treated with liso-cel with month-3 CR included *ICOS*, *CD28*, *CD40LG*, and *KLRB1* in TRANSCEND NHL 001 cohort [46]. The relative expression of mRNA for costimulatory molecules (ICOS and 4-1BB) was significantly higher for the CR patients in the relma-cel trial [59].

The assessment of TME dynamics after CAR-T infusion showed a significant shift in the immune checkpoint expression profile and its association with response. Multiplex immunohistochemistry in axi-cel cohort post-infusion showed that durable responses were associated with a significant increase in the density of PD-1+TIM3+LAG3- cytotoxic T-cells [67]. Significant transcriptomics changes in tumor biopsies from pre-treatment to 2 weeks after axi-cel with the upregulation of immune checkpoint encoding genes (CD274, CD276, and CTLA-4) were also associated with response to therapy [61].

The data regarding the role of immune checkpoints in the tumor microenvironment for bispecific antibody therapies is rather scarce. The expression of PD-1 and LAG-3 on CD8 T-cells within the baseline tumor biopsies of patients with DLBCL was assessed using immunofluorescence in the ELM-1 cohort of patients treated with odronextamab. While the proportion of CD8 T-cells that expressed PD-1 and/or LAG-3 was numerically higher among the patients with response, this difference was not statistically significant [51]. A gene expression signature analysis of the baseline tumor samples of patients receiving glofitamab treatment demonstrated significantly lower PD-1-high T-cell signatures in complete responders versus patients with the progression of the disease [49].

A notable yet unexpected observation in patients treated with odronextamab was the significantly higher density of PD-L1+ cells within baseline tumor biopsies among the responders compared to non-responders. Moreover, an increase in PD-L1+ cells from baseline to Week 5 was noted across patients with DLBCL, suggesting that PD-L1 expression may be an adaptive increase in interferon signaling as a response of the tumor to immune engagement by the therapy [51].

Studies on immune checkpoint expression in T-cell redirecting therapy highlight the complexity of interpreting these markers, which can indicate either T-cell activation or exhaustion depending on their co-expression and TME context. Responses to treatment with CAR-T were associated with a tumor microenvironment that promotes T-cell activation, supported by elevated levels of costimulatory molecules such as ICOS and 4-1BB. The data regarding these characteristics for BSAs is lacking. The expression of inhibitory ligands, particularly PD-L1 either by tumor or TME macrophages, demonstrated a negative influence on response and prognosis for patients treated with CAR-T. The difference in the prognostic significance of high PD-L1 density observed in patients treated with odronextamab is intriguing as it may indicate distinct mechanisms of resistance in patients treated with BSAs, but should be confirmed in other cohorts in future studies.

## 5. Stromal Components, Adhesion Factors, and Cytokines

Studies of the stromal components and their prognostic role in T-cell redirecting therapies are limited, especially those utilizing immunohistochemistry. Most studies have instead focused on stromal gene expression as part of comprehensive gene expression profiling. Gene expression profiles from the biopsy samples of patients with relapsed/refractory LBCL from the ZUMA-7 trial identified a distinct group of tumors enriched by stroma, myeloid and endothelial cells, *NOS2*, *TGF-β*, *B7-H3*, *ARG1*, and hypoxia markers. Notably, this cluster correlated negatively with the event-free survival of patients undergoing treatment with axi-cel [54].

Previously, G. Lenz et al. analyzed biopsy samples from untreated patients with DLBCL. Their gene expression profiling revealed two distinct gene signatures unrelated to malignant cells. The “stromal 1” gene signature was enriched in genes encoding extracellular matrix proteins (fibronectin, osteonectin, collagens, and laminins) or enzymes involved in the synthesis and remodeling of matrix components (collagen synthesis modifiers, metalloproteases, and connective tissue growth factor) and the antiangiogenic factor thrombospondin. This cluster was positively associated with survival after CHOP or R-CHOP therapy. The “stromal 2” gene signature had the opposite effect on the prognosis. This cluster included genes for endothelial cells and angiogenesis regulators [48]. Building on these findings, the subsequent gene expression profiling of baseline tumor biopsies from patients treated with liso-cel, using the gene sets identified by Lenz et al., demonstrated that patients who maintained a complete response at 3 months post-CAR-T infusion exhibited a strong expression of the “stromal 1” gene signature [46].

In the relma-cel study, the expression levels of tumor-associated fibroblast markers (*AP*, *TNC*, *CSPG4*, *PDGFRA*, *S100A4*, *ASPN*, *STC1*, and *ITGAM*) were higher in the patients with PR than with CR [59].

The capacity of the TME to support T-cell infiltration is influenced by the endothelial state, extracellular matrix architecture, and the profile of chemokines and cytokines, facilitating lymphocyte access and migration to tumor loci. The analysis of biopsies of patients treated with relma-cel demonstrates that in patients with complete response to CAR-T, CD3+ T-cells were distributed within the tumor, whereas in partial responders, characterized by high levels of tumor-associated macrophages, T-cells were largely excluded from the tumor loci [55]. However, the studies of the mechanisms of this trafficking were limited to the analysis of gene expression profiles in different panels. Based on the data of the RNA sequencing of the biopsy samples of patients before the relma-cel infusion, in complete responders, the expression of cytokines genes *CCL2*, *CCL3*, *CCL4*, *CCL5*, *CXCL8*, *CXCL9*, *CXCL10*, *CXCL11*, *CXCL12*, *CXCL16*, *IL-10*, and *TGF-β* was lower compared with patients who achieved only partial response. The expression of *CCR6*, *CCR10*, *CXCR3*, and *CXCR4* genes was increased in patients with CR [55]. In tumor samples, *IL-8* gene expression was elevated in patients who failed to respond or relapsed after axi-cel [72]. In the independent cohort of patients receiving CAR-T therapy, on-treatment fine needle biopsies were analyzed using single-cell sequencing, demonstrating that high CCL8, CCL13, or CCL18 levels in myeloid cells were linked to poor outcomes after CAR T-cell therapy for LBCL [74].

Mutations in the gene encoding the adhesion protein CD58 (ligand of CD2) were shown to be more common in patients progressing after CAR-T than responders [75], and were an unfavorable prognostic factor. Patients with LBCL with aberrations in CD58 had lower PFS during CAR-T therapy by axi-cel [76,77].

Thus, the available information on the prognostic influence of stromal components, the profile of cytokines, chemokines, and adhesion molecules on the effectiveness of CAR-T therapy is extremely limited. Regarding BSAs, there is currently no information assessing the impact of stromal components, adhesion factors, and cytokines on therapy effectiveness or patient prognosis. However, insights from CAR-T therapies suggest that the mentioned factors may play a role in treatment outcomes, making them valuable targets for further investigation in the context of BSAs. Furthermore, while gene expression profiling provides valuable insights, it only partially addresses the complexity of T-cell migration and interaction with the tumor. Critical aspects such as properties of the endothelial barrier, spatial assessment of chemokine gradients, and the physical and mechanical properties of the matrix remain unexplored. These factors are crucial for understanding the full spectrum of mechanisms governing T-cell trafficking from the bloodstream into the tumor microenvironment, suggesting a significant gap in our current methodological approaches and emphasizing the need for comprehensive studies that would expand our understanding of T-cell redirecting therapies.

## 6. Challenges and Opportunities

While the potential applications of CAR T-cell therapies and BSAs extend well beyond hematological malignancies, our review focuses specifically on the clinical studies of anti-CD19 CAR T-cells and anti-CD3/CD20 BSAs approved for r/r LBCL. We acknowledge that this focus on approved products for B-cell lymphomas limits our scope and may not fully capture the published data on the TME correlation with the outcomes of CAR-T and BSA therapies. A key limitation of our analysis is the reliance on a small number of patient data and cohorts, primarily drawn from registrational trials such as ZUMA-1 and ZUMA-7 for axi-cel, TRANSCEND NHL-001 for liso-cel, the JULIET pivotal trial for tisa-cel, and a small relma-cel cohort. Moreover, the same patient populations often underpin multiple reports published at different times, which might lead to overlapping or contradictory findings. The dataset is even more limited for BSAs. Furthermore, the methodologies employed in these studies vary significantly, most encompassing bulk gene expression profiling and immunofluorescence utilizing diverse marker panels. This variability complicates the comparison of the results across different studies and can lead to contradictory findings. Another significant limitation is the focus of most studies on immediate response metrics like complete response or partial response, and objective response rate, with less emphasis on long-term outcomes such as progression-free survival and even less reporting the influence on overall survival. 

The analysis revealed several research gaps that could be addressed to advance the field of T-cell redirecting therapies for LBCL, as well as some insights from CAR-T studies providing valuable perspectives for BSAs. These include a more comprehensive analysis of immune cell density and composition, particularly in BSA treatments, to understand not just the quantity but also the functional state of cells like macrophages and tumor-infiltrating lymphocytes. This suggests the use of standardized panels like Immunoscore and Immunosign21. The role of macrophage polarization (M1 vs. M2) in prognosis and therapy outcomes also needs more exploration. To identify the M1 polarization of TAMs, a panel may be used that evaluates membrane expression of CD80, CD86, iNOS, and MHC-II, and for M2–CD206, CD163 as well as a number of intracellular factors: STAT6, IRF4, JMJD3, PPARδ/γ, c-maf, and c-myc [78]. Future studies should assess polarization states to better understand their impact on therapy efficacy. There is a need for an expansion of immune checkpoint analysis to evaluate comprehensive profiles that include activating checkpoints in BSAs, such as ICOS, CD28, 4-1BB, CD40L, and KLRB1, as they showed to be significant in CAR-T cohorts. Besides assessing isolated markers, studies should evaluate comprehensive profiles that distinguish between activated and exhausted T-cells. Candidate markers for such panels may be PD-1, TIM-3, LAG-3, or TOX-1 as was demonstrated by Scholler et al. [61]. Studies on the immune checkpoint ligands need to expand beyond PD-L1 and may include MHCII, B7-H3, B7-H4, IDO1, Galectin-9, CD155, and VISTA for a more nuanced understanding of TME immune interactions. Techniques such as immunofluorescence or immunohistochemistry are needed to explore the spatial characteristics of tumor architecture and checkpoint distribution. The prognostic value of stromal gene expressions, such as those associated with the extracellular matrix, also remains underexplored in both the CAR-T and BSA contexts. In the relma-cel study, data were provided on the effect of the gene expression of tumor-associated fibroblasts (*AP*, *TNC*, *CSPG4*, *PDGFRA*, *S100A4*, *ASPN*, *STC1*, and *ITGAM*) and cytokines (*CCL2*, *CCL3*, *CCL4*, *CCL5*, *CXCL8*, *CXCL9*, *CXCL10*, *CXCL11*, *CXCL12*, *CXCL16*, *IL-10*, and *TGF-β*) and their receptors (*CCR6*, *CCR10*, *CXCR3*, and *CXCR4*) on the probability of achieving CR [59]. However, the cohort of patients analyzed was small. Therefore, further studies of the above markers are promising not only in relation to CAR-Ts, but also to BSAs. In the treatment with BSAs, the role of the adhesion molecule CD58, which has already demonstrated its negative effect in anti-CD19 CAR-Ts, has not been studied. A deeper understanding of these elements could reveal new therapeutic targets and prognostic markers. Finally, integrating advanced technologies like genomic, proteomic, and imaging technologies could enhance the accuracy of TME assessments. This comprehensive approach would help to fill the significant gaps in our current methodological approaches and improve the effectiveness of T-cell redirecting therapies in LBCL.

## 7. Conclusions

**Figure 1:**
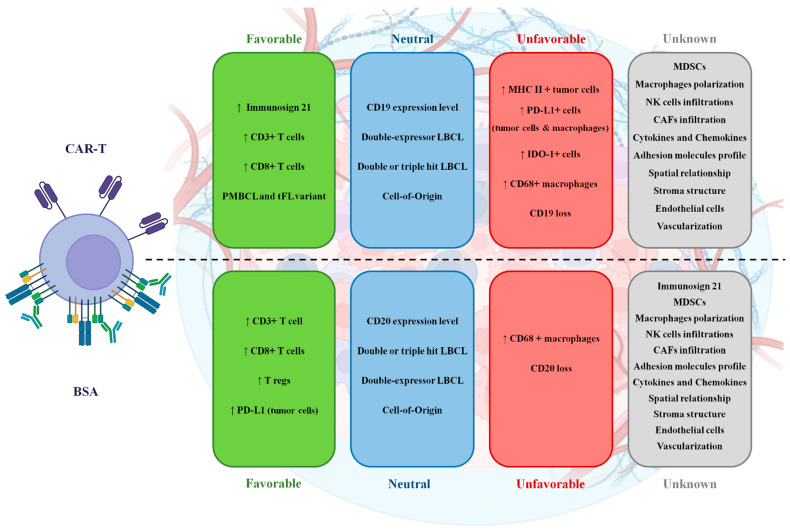
The influence of LBCL tumor microenvironment components on the efficacy of CAR-Ts (**top**) and bispecific antibodies (**bottom**). Favorable: Factors associated with an increase in clinical efficacy endpoint parameters, including overall response rate, progression-free survival, event-free survival, and overall survival. Neutral: Factors shown to have no association with clinical efficacy endpoint parameters. Unfavorable: Factors associated with a decrease in clinical efficacy endpoint parameters. Unknown: Factors that were not assessed in the analyzed reports, or where contradictory findings hinder conclusions regarding their role.

The role of the tumor microenvironment in T-cell redirecting therapies such as CAR-Ts and bispecific antibodies has proven pivotal yet complex, underscoring a nuanced interplay between tumor biology and therapeutic efficacy in LBCL. As illustrated in Figure 1, the variability in TME composition—ranging from immune cells to stromal elements—critically influences patient outcomes, highlighting a significant area for deeper investigation. The existing literature, primarily from a limited number of trials and using diverse methodologies, provides a foundational understanding but also indicates substantial gaps in comprehensively mapping TME dynamics and their therapeutic implications. Future research should aim to standardize assessment methods and expand studies to include a broader range of TME components, which may unveil new prognostic markers and therapeutic targets. Ultimately, advancing our grasp of the TME’s role will enhance the strategic development of T-cell therapies, potentially leading to more robust and sustained responses in LBCL patients.
